# Historical Human Footprint on Modern Tree Species Composition in the Purus-Madeira Interfluve, Central Amazonia

**DOI:** 10.1371/journal.pone.0048559

**Published:** 2012-11-20

**Authors:** Carolina Levis, Priscila Figueira de Souza, Juliana Schietti, Thaise Emilio, José Luiz Purri da Veiga Pinto, Charles R. Clement, Flavia R. C. Costa

**Affiliations:** 1 Programa de Pós-Graduação em Ecologia, Instituto Nacional de Pesquisas da Amazônia (INPA), Manaus, Amazonas, Brasil; 2 Programa de Pós-Graduação em Botânica, Instituto Nacional de Pesquisas da Amazônia (INPA), Manaus, Amazonas, Brasil; 3 Coordenação de Biodiversidade, Instituto Nacional de Pesquisas da Amazônia (INPA), Manaus, Amazonas, Brasil; 4 Coordenação de Tecnologia e Inovação, Instituto Nacional de Pesquisas da Amazônia (INPA), Manaus, Amazonas, Brasil; New York State Museum, United States of America

## Abstract

**Background:**

Native Amazonian populations managed forest resources in numerous ways, often creating oligarchic forests dominated by useful trees. The scale and spatial distribution of forest modification beyond pre-Columbian settlements is still unknown, although recent studies propose that human impact away from rivers was minimal. We tested the hypothesis that past human management of the useful tree community decreases with distance from rivers.

**Methodology/Principal Findings:**

In six sites, we inventoried trees and palms with DBH≥10 cm and collected soil for charcoal analysis; we also mapped archaeological evidence around the sites. To quantify forest manipulation, we measured the relative abundance, richness and basal area of useful trees and palms. We found a strong negative exponential relationship between forest manipulation and distance to large rivers. Plots located from 10 to 20 km from a main river had 20–40% useful arboreal species, plots between 20 and 40 km had 12–23%, plots more than 40 km had less than 15%. Soil charcoal abundance was high in the two sites closest to secondary rivers, suggesting past agricultural practices. The shortest distance between archaeological evidence and plots was found in sites near rivers.

**Conclusions/Significance:**

These results strongly suggest that past forest manipulation was not limited to the pre-Columbian settlements along major rivers, but extended over interfluvial areas considered to be primary forest today. The sustainable use of Amazonian forests will be most effective if it considers the degree of past landscape domestication, as human-modified landscapes concentrate useful plants for human sustainable use and management today.

## Introduction

The common belief that natural environments of the Americas were relatively untouched by humans before the European conquest is no longer accepted [1], [2]. Starting at least 3000 years before present, pre-Columbian populations increased in size, density and duration of their occupations [3]. Landscapes and many plants were domesticated in different degrees to sustain these societies [4]. The extent and degree of Amazonian landscape domestication, however, are still controversial. While some archaeologists suggest extensive modification of the landscape [5], [6], ecologists often argue the opposite, that most of the Amazon basin shows few signs of disturbance [7], [8]. Bush & Silman [9] and McMichael *et al.* [10] suggest an intermediate hypothesis, in which the intensity of human impacts decreases exponentially with increasing distance from the major Amazonian rivers, especially in non-seasonal forests. Human impacts that are easily recognized include changes in soils and relief, such as anthropogenic soils and geoglyphs, as well as changes in forest composition, often recognized as anthropogenic forests [11].

Anthropogenic soils, called *Terra Preta de Índio* or Amazonian Dark Earth (ADE), are usually found on bluffs along the major rivers [12], [13]. This led to the hypothesis that pre-Columbian settlement in Amazonia was mostly located on the bluffs of white water rivers [12]. The preference for these sites is explained by the concentration of food resources and more fertile soils in the adjacent floodplains [12]. Nevertheless, hundreds of geoglyphs have been found in the upper Purus-Madeira interfluve distributed over an area 250 km from south to north, encompassing both floodplains and interfluvial uplands [14]. In the Llanos de Mojos in Bolivian Amazonia most of the earthworks are on interfluves in a forest-savanna mosaic with many useful species [15]. The occurrence of these earthworks suggests the existence of complex societies and dense populations in interfluvial areas, environments previously described as unable to support large numbers of people [12], [14]. Aside from the geoglyphs, archaeological studies are rare on the interfluves, often thought to constitute the largest proportion of the Amazonian landscape [8]. This notion of hard-to-occupy interfluves requires caution, however, as the Amazonian landscape contains numerous drainage basins of rivers and streams that extend into the interfluves and are easily accessed. These seasonal and permanent wetlands represent more than 30% of the basin [16], so aquatic resources are often easily available.

Upon identifying useful species in the forest community, Native Amazonians frequently increased their abundance, creating oligarchic forests often associated with ADE [11], [17]. These forests, known as anthropogenic forests, are dominated by one or more useful species due to human activity [11], but are commonly considered primary forests because of their diversity, stature, and closed canopies. In most cases, the only way to identify signs of past manipulation is by assessing the distribution and abundance of useful species [18]. Forest patches dominated by Brazil nut trees (*Bertholletia excelsa*), known as *castanhais*, and by the palm caiaué (*Elaeis oleifera*), known as *caiauezais*, are well-known anthropogenic forests [11], [19]. Brazil nut trees with diameters greater than 220 cm are probably older than the colonization of Amazonia, counting from the establishment of Belém, Pará, in 1616 [20]. As caiaué is no longer intensely used by traditional communities, *caiauezais* can be considered pre-colonization also [21].

The evidence left by Native Amazonian populations reflects different degrees of landscape domestication practiced in pre-Columbian times [4]. In settled and cultivated landscapes, signatures can be found in the soils, such as ADE, charcoal and crop phytoliths, which may extend for several kilometers from the river edge. Around cultivated landscapes are managed anthropogenic forests, which extend further away. Managed landscapes have different human footprints, with little or no charcoal and crop phytoliths, but with many useful plants [18]. Further away the forests are used primarily for gathering and hunting. These forests are often promoted in the sense that hunter-gatherers often discard seeds along trails or may even plant seeds or cuttings at preferred sites, as reported for the Kayapó in southeastern Amazonia [11], [22]. Since hunters and gatherers visited essentially all of Amazonia outside the settled, cultivated and managed landscapes [8], these promoted landscapes probably occupy most of the basin, but their identification requires botanical and ecological techniques, such as those used here, rather than the archaeological methods advocated by Bush & Silman [9] and McMichael et al. [10].

In this study, we examine past human modifications of the forest from the Solimões, Purus or Madeira Rivers into their interfluve, considering also the role of secondary rivers. The environmental conditions of these major rivers are favorable for human settlement and numerous ADE sites have been found on their riverside bluffs, especially along the Solimões and Madeira [13]. In many areas, the interfluvial forests are exposed to flooding during the rainy season [16], creating environments unsuitable for year-round human occupation and intensive agriculture. Even under these conditions, we show that interfluvial forests have signs of manipulation at different distances from rivers.

We assessed human intervention in the forest by the abundance, richness and basal area of useful arboreal species, mostly fruit trees and palms, and studied the hydrological conditions of the sites as a possible ecological factor influencing the distribution and abundance of useful palms. We considered the mass of charcoal in the soil of each site as another indication of past human cultivated landscapes [9], [10]. Fire was the most powerful tool for landscape transformation [6] and the presence of abundant charcoal in the soil is an important evidence of human disturbance in tropical forests [9], [23]. Due to the lack of archaeological data on the interfluve, we mapped archaeological evidence (ADE), and two types of anthropogenic forests, *castanhais* and *caiauezais*, around the study sites. These data were used to test the hypothesis that human modifications in forest landscapes decrease with distance from rivers, as suggested by Bush & Silman [9] and McMichael *et al.* [10].

## Results

### Archaeological evidences

**Figure 1:**
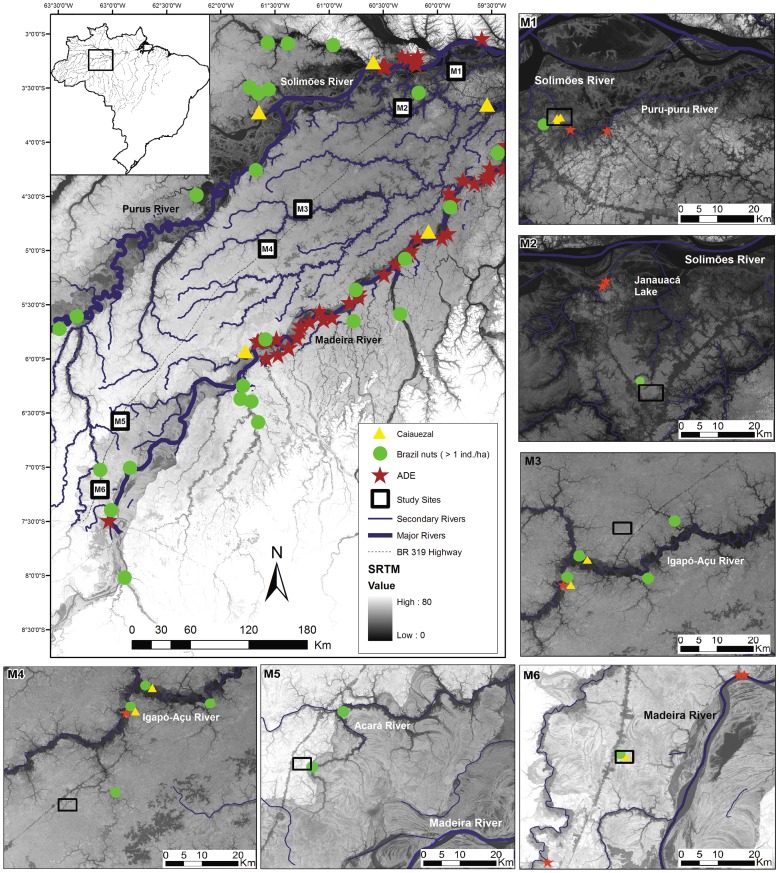
Map of the study area. The large map shows the central-northern Purus-Madeira interfluve. The empty squares are the six sampling sites at different distances from major rivers. In each site, two plots were sampled for floristic analysis and five for charcoal analysis. Yellow triangles are *caiauezais*, green circles are *castanhais* (Brazil nuts >1 individual/hectare) and red stars are ADE (Amazonian Dark Earth sites). ADE data from WinklerPrins and Aldrich [13], *castanhais* from RADAMBRASIL [24] and *caiauezais* from Moretzsohn *et al.* [25]. The other six maps show the newly identified Amazonian Dark Earth sites (ADE) and anthropogenic forests in the vicinity of each site, identified by interviews with local residents or by observation.

Archaeological sites and anthropogenic forests were found inside and around the six study sites, far from major rivers (Fig. 1). All sites with ADE were on the banks of secondary rivers (>50 m wide) and had not previously been identified, e.g., WinklerPrins and Aldrich [13]. *Castanhais* and *caiauezais* are mostly near secondary river banks, but also occurred in the interior; for more information about archaeological evidence and anthropogenic forests see Text S2. In the sites closest to main rivers, there were shorter distances between the archaeological evidences and the sampling plots (Table 1), and more evidences in general, as expected from the hypothesis. These results highlight the need for more field archaeological investigation in this and other interfluves to continue testing the hypothesis.

**Table 1:** Means and standard deviations of all botanical data and distances measured in the six study sites along the Purus-Madeira interfluve, Amazonas, Brazil.

	M1	M2	M3	M4	M5	M6
Abundance of all useful species^1^	195,5±36,5	131±13	75±19	42	69,5±10,5	132,5±5,5
Total abundance	520±39	614±39	661,5±1 28,5	588	551,5±41,5	531,5±76,5
Abundance of useful palms	133,5±27,5	84±26	19± 3	8	28±4	85,5±2,5
Abundance of useful dicotyledonous trees	62±9	47±13	56±22	34	41,5±6,5	47±3
Basal area of all useful species^2^ (m^2^/ha)	7,47±1,64	3,6±0,14	2,40±0,39	1,67	3,67±0,27	7,16±2,74
Total basal area (m^2^/ha)^3^	19,99±2,03	22,27±0,31	23,37±2,01	26,92	24,22±2,67	25,35±3,13
Richness of all useful species	9±1	12±1	14	10	11,5±1,5	12±1
Total richness	44,5±4,5	142±9	164,5±4,5	164	76±1	90±2
Distance from main rivers (km)	11±0,5	36	91,5±0,5	80	39,5±0,5	18,5±0,5
Index of rivers distances	[-]0,06±0,04	0,58±0,03	0,87±0,01	0,93	0,79±0,02	0,77
Distance from ADE (km)	5±0,7	33,0	23	31	-	37
Distance from anthropogenic forests (km)	0,5	3,0	13±1,4	15	3±0,7	0,5±0,7

1 The values of species abundance are the number of all useful trees and palms with DBH≥10 cm in 1 ha.

2 The value of species basal area are the basal area of all useful trees and palms with DBH≥10 cm in 1 ha.

3 The values of species richness are the sum of all trees and palm species with DBH≥10 and <30 cm in 0.5 ha and the largest in 1 ha plot.

### Charcoal in the soil

**Figure 2:**
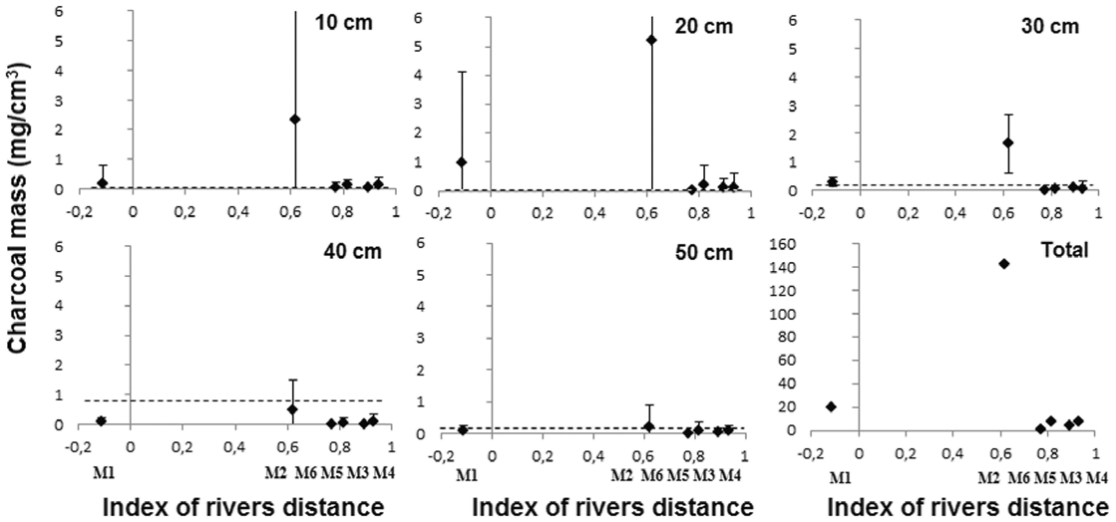
Mass of charcoal in the soil (mg/cm^3^) for each soil layer from 10 cm to 50 cm in depth. The plots are in order of increasing distance from the sites to the rivers, expressed by the index of rivers distances. Each point is the average mass of charcoal in the soil from 14–15 samples in each area. The vertical lines represent the standard deviation. The dotted line represents the median value of the mass of charcoal found at each depth in the soil of forests north of Manaus in Central Amazonia, which is the value expected in soils without past human activities [26].

Macroscopic charcoal particles were recorded in all areas and in all soil layers down to 50 cm (Fig. 2). Only in the 30–40 cm layer at site 6 was charcoal absent. Higher values of charcoal were detected in the sites closest to major and secondary rivers. At M1 the charcoal mass was high in the top 20 cm of soil. Charcoal particles were abundant in all layers at M2, 36 km from the main river, and the mean was much higher than the median value observed by Piperno and Becker [26] in upland forest soils 90 km north of Manaus in Central Amazonia, which is thought to be the value expected in soils without past human activities. All other sites had charcoal close to the median value.

### Relationship between the useful tree community and the distance from rivers

**Figure 3:**
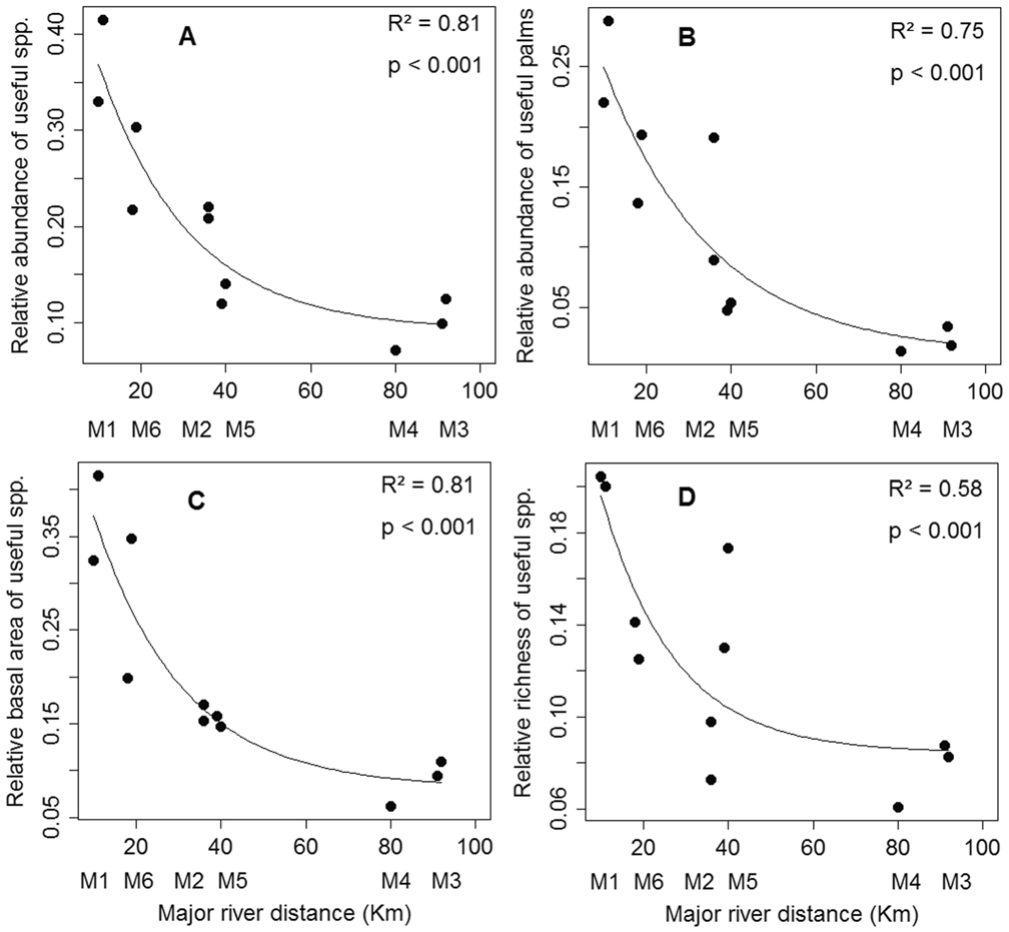
Relationships between useful tree parameters and the distance to major rivers. Relationships between the useful trees parameters and the distance to the relative abundance of useful species per plot (A), the relative abundance of useful palms per plot (B), the relative basal area of useful species per plot (C) and the relative richness of useful species per plot (D), with the distances from major rivers. Points are the plots of all sites, totaling 11 plots. The shortest straight line distance in km from the plot to the Solimões, Purus or Madeira River was evaluated.

The relative abundance, basal area and richness of useful trees and palms decreased with distance from major rivers (Fig. 3). The relationship between these variables was a highly significant negative exponential curve. Plots located from 10 to 20 km from a major river had 20–40% useful tree and palm species, plots with distances between 20 and 40 km had 12–23%, plots more than 40 km had less than 15%. In the first 20 km from major rivers there was a rapid decrease in useful tree species and individuals. Beyond 40 km, the proportions of useful individuals and species decreased slowly. The sites with higher concentrations of useful plants (M1, M2 and M6) were on *paleo-várzeas*, pre-Holocene floodplains [24], [27] (Table 1). These higher abundances are not of single species, but suites of useful species that vary among sites (Table S1).

**Figure 4:**
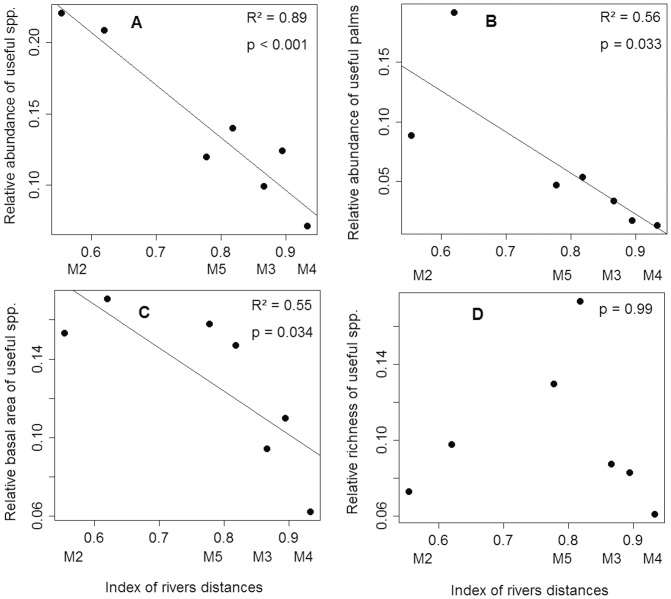
Relationships between useful tree parameters and the distance to secondary rivers. Relationships between the relative abundance of useful species per plot (A), the relative abundance of useful palms per plot (B), the relative basal area of useful species per plot (C) and the relative richness of useful species per plot (D), with the distances from secondary rivers. Points are the plots of sites M2, M3, M4, M5, totaling seven plots. The index of rivers distances is the sum of the inverse distances from each plot to all perennial rivers greater than 50 m wide in a 25 km diameter zone around the sites.

In order to focus on the influence of the secondary rivers, we analyzed the relationship between useful tree parameters and the distance to secondary rivers, excluding plots from the two sites closest to the main rivers, M1 and M6. We observed a negative relationship between abundance and basal area of useful trees and the index of distance from secondary rivers crossing the interfluve (Fig. 4). This relationship was linear and explained ≥50% of the variance. This analysis showed that the abundance and the basal area of useful species in areas away from major rivers is closely related to their distance to secondary rivers, suggesting that the secondary rivers were also occupied by pre-modern populations.

### Palms

**Figure 5:**
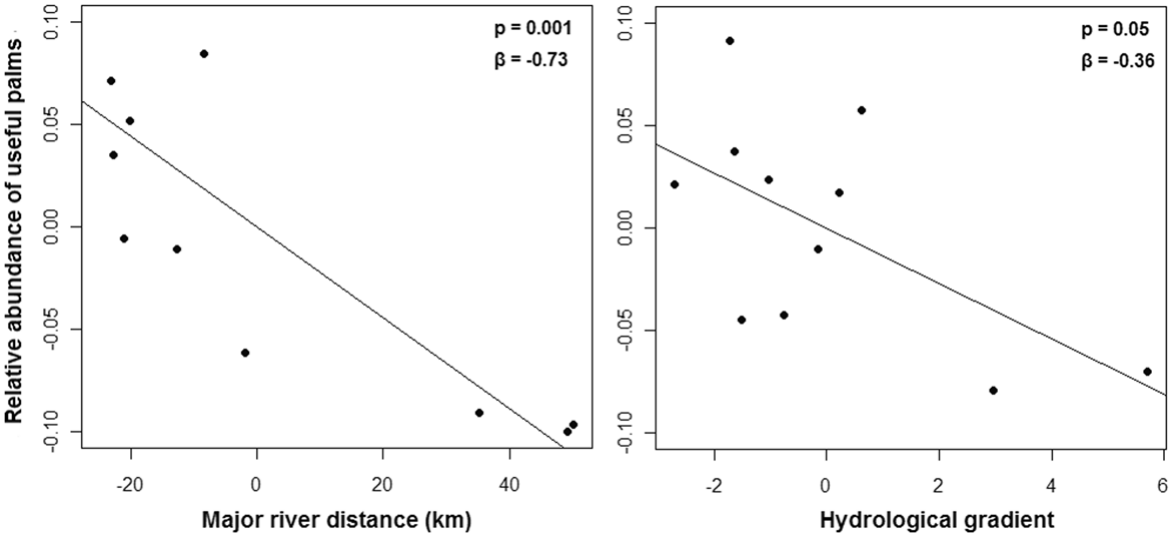
Multiple regression analysis of useful palms and the distance from rivers and the hydrologic gradient. Partial regressions between the relative abundance of useful palms and the distance of each plot to the major rivers (left) and the hydrologic gradient (right). The full multiple regression model has an *R^2^* = 0.73. Points are the plots of all sites, totaling 11 plots.

Palms were the most abundant useful family in all plots (Table 1, see also Table S1). The relationship between the relative abundance of palms and the distance to rivers was as strong as the relationship for all useful species together (Fig. 3B and 4B). Excluding the useful palms, the relationship between the abundance of dicotyledonous trees and the distance to major rivers was less significant (p = 0.05). A multiple regression analysis of the relationship between the relative abundance of useful palms, the distance from rivers and the hydrologic gradient indicated a strong negative effect of the distance from rivers (Fig. 5) and a significant negative effect of the hydrologic gradient on the abundance of useful palms (Fig. 5). The hydrologic gradient was not the major determinant of useful palm abundance in the interfluve, and a large fraction of variance is attributable to distance per se, which is our proxy for anthropogenic effects.

## Discussion

### Human intervention in the landscape decreases with distance from rivers

Our data support McMichael *et al.*'s [10] hypothesis and the expectations of Bush & Silman [9] that human intervention in the landscape decreases with distance from major and secondary rivers. However, the extent of past human impact in the forest observed in our study is much higher than expected by these authors and the assumptions of Peres *et al.* [7] and Barlow *et al.* [8]. Using only simple regressions with distance to major and secondary rivers, which reflect the distance from possible pre-Columbian settlements, we explained 50–90% of the variation in the useful tree parameters. We found high abundances of useful tree species up to and beyond 20 km into the interfluve, and also the presence of anthropogenic forests and ADE far from the main rivers, but close to secondary rivers. Studies that only assessed past human disturbance in terms of charcoal, pollen and phytoliths of cultivated plants [9], [10] failed to detect signals from less intensive interventions in the landscape, such as enrichment of forest through extractive activities and hunting. Less intensive activities also caused changes in the concentration of useful plants in the past [18], [28] and even today contribute to increases in the concentration of certain plants, such as Brazil nut, along trails [22], [29].

Archaeological evidence and useful species composition found at M1, the site closest to the major rivers, indicate forest management practices by different groups in different historical moments. The landscape surrounding site M1 includes numerous rivers and lakes (Fig. 1) that were and still are waterways used for movement and fishing. The existence of ADE, *castanhais* and *caiauezais* near plots are evidence of landscape domestication by indigenous groups before European conquest. On the other hand, all inventoried individuals of the rubber tree (*Hevea brasiliensis*) had marks of extraction. Rubber is usually rare in the forest [30]; however, we found 30 individuals in a one ha inventory (Table S1), the density of a very common species. Hence, some of these forests were reoccupied, exploited and transformed by rubber tappers in the early twentieth century, resulting in the increased abundance of rubber trees (and possibly other useful species) in the forest.

Distinguishing between pre-Columbian and post-conquest management events requires more historical and ethnographic studies in each locality. Except at M1, we didn't find signs of rubber tapper impacts. However, in 1970 the BR-319 Highway was constructed, allowing movement of modern migrants into the interfluve. Most local residents in the vicinity of the study sites have been there since this period. Current management practices in mature forest performed by these recent arrivals probably wouldn't significantly affect the tree community with DBH≥10 cm.

We expect that the footprints of past human activities in the forest will be found in other parts of Amazonia given results in other areas of the world. Around ancient Roman ruins in France, the composition and diversity of plants reflects the impacts of agriculture 1500 years after abandonment [31]. In the Maya forests in Central America, past human management of useful species was identified 1000 years after this civilization's decline [32]. In Central Africa, current tree species composition and diversity still reflects human disturbances after nearly four centuries [33]. Our results showing a gradient of human manipulation in the forest from the major rivers into the interfluve agree with these other studies, however, our study is preliminary and more field investigations that assess forest composition are needed to fully understand variation in landscape domestication across Amazonia.

It is too early to make basin-wide projections, such as Barlow *et al.*'s ([8], p. 4) suggestion of “a largely imperceptible footprint from subsistence hunting and resource extraction across vast tracts of Amazonian forests”. This caution is especially true for the interfluves, all of which are insufficiently sampled [10], [34]. Only the Tapajós-Xingu interfluve has a large number of ADE records [13], [35], but the others have not been adequately surveyed. In the Purus-Madeira interfluve, we detected ADEs near black water secondary rivers and even in places susceptible to flooding in anomalous years. Geoglyphs were found in the upper Purus-Madeira interfluve [14], indicating that this region is unique and may not be representative of other interfluves. Moreover the abundant social, cultural, historical and ecological variation observed across the Amazon basin makes difficult to extrapolate our results to broad regional patterns. On the other hand, all of these observations suggest that if we look, we will find more and more evidences of past human activities on the interfluves.

### Ecological factors and past human management influence useful tree and palm abundance and distribution

Ecological conditions can explain tree mono-dominance without invoking the need for human dispersal, especially of some palm species, e.g., *Mauritia flexuosa* in swamp forests [36]. A gradient of hydrological conditions runs from the floodplain to the interfluve, and in wetlands the flood-level is a determinant of plant distribution along this gradient [37]. Even in upland forests, some palm species respond to the hydrological condition of soils [38]. As expected, we found a significant effect of the hydrologic gradient on the abundance of useful palms in our multiple regression model. However, the effect of distance to rivers, after partitioning out the hydrologic gradient effect, was stronger, probably due to past human management.

If ecological conditions were the sole determinants of plant distributions in the interfluve, we should find roughly the same useful tree and palm communities on *paleo-várzeas*, which are pre-Holocene floodplains with similar geomorphological and hydrological conditions. Instead, we found unusual species-environment associations: *Bertholletia excelsa*, *Attalea speciosa*, which are naturally associated with upland forests, were found as dominants in M6, a site on the *paleo-várzeas* of the Madeira River (see also Table S1). In addition, in Central Amazonia, *Euterpe precatoria* and *Oenocarpus bataua* occur mainly in low areas with poorly drained soils [38]. However, we found these two species in the same plots with *Attalea maripa* and *Theobroma* spp. (Table S1), which are disturbance indicators and associated with archaeological sites [11], [39], [40] normally found in non-flooded areas [41]. The co-occurrence of these species, therefore, is likely to be due to their usefulness to humans. Considering that *Oenocarpus bataua* and *Attalea maripa* are usually more abundant in forests with more open canopies [42], [43], the high density of these species at M2 and the presence of abundant charcoal particles in this area are probably associated with the historical presence of humans and fire in the region, which may have increased light penetration in the forest.

We observed forests dominated by a number of useful species with different environmental preferences, including species occurring outside of their natural environments. Thus, ecological conditions alone can't explain useful tree and palm dominance and distribution in the interfluve; pre-conquest and historical management must be considered also. Our results suggest that pre-Columbian populations of the interfluve affected the distribution and abundance of palms even in places that were not ideal for their establishment.

### Charcoal and landscape modification

Paleoecological analyses in Amazonia found large amounts of charcoal associated with pollen of cultivated plants, indicating that fire was associated with past agricultural practices [44]. Charcoal, pollen and phytoliths of cultivated plants are more direct evidences of past agricultural practices than the modern useful arboreal community. We found charcoal in the soils of all areas at almost all depths, indicating that interfluvial forests were burnt at different moments in the past. If charcoal particles in the top 20 cm soil depth are often of modern origin [45], most of the charcoal at depths greater than 20 cm is probably pre-Columbian. Charcoal is common in soils of the interfluve; however phytoliths from agriculture and early successional growth taxa related to human disturbance were found to be scarce in the interior of the interfluve [45].

Despite the widespread occurrence of charcoal in the landscape, its abundance was high in only two sites (M1 and M2). The forest in M1 was used by rubber tappers, so the charcoal in the top 20 cm soil depth may be related to their activities. At M2 we observed even larger amounts of charcoal in all soil layers. This site is located 36 km from the Solimões River and 5 km from the Janauacá River, a wide river stretching into the center of this portion of the interfluve (Fig. 1). We also found high densities of useful species in these plots (Table 1). All this evidence of landscape domestication is indicative of intensive agricultural and other management activities in these two areas, even though we did not study pollen or phytoliths.

Since not all charcoal particles found in Amazonian forests can be attributed to past human intervention [26], the charcoal particles we found in other sites of the interfluve may be from natural fires or low impact human activities. Low intensity fires without another indication of intensive clearing are probably not a signal of extensive forest disturbance. In two of 13 sites studied on the Purus-Madeira interfluve, charcoal was found associated with phytoliths, confirming human agricultural activities at these sites, but little clearing of the interfluve forest in other sites far from rivers [45]. Our results also suggest that not all sites on the interfluve were affected by human fire management; however, further archaeological studies are needed for a more complete understanding of the locations of the pre-Columbian cultivated landscapes within this interfluve.

Charcoal analysis is not useful to detect human activities not related to fire, such as planting useful species on trails inside the forest [22] and discarding seeds while walking to extract fruit or while hunting [18], [28], [46]. Hunter-gatherers can travel 25–26 km from their settlement on a hunting trip [47], [48], essentially putting all of Amazonia within the category of hunted landscapes [8] where landscape management is likely to happen. Stahl [49] reviewed Amazonian studies that demonstrate how the cumulative historical impact of small bands of hunter-gatherers is probably greater than that of traditional farmers, although the latter also manipulate the forests near their settlements. Contemporary hunter-gathers are sophisticated and dynamic managers of forest landscapes, subtly thinning some areas to make space for their preferred resources and creating “wild orchards” [50]. They exploit temporary gaps, disperse fruit trees and palms, and move their camps regularly, concentrating resources at preferred locations over wide areas of forest landscapes. Over thousands of years the results are dramatic, but the forests look mature, untouched, pristine to the untrained eye. Even though we did not find a considerable mass of charcoal in M6, useful species composition suggests a history of intense human management at this site, like that reviewed by Stahl [49]. *Attalea speciosa*, *Astrocaryum aculeatum* and *Bertholletia excelsa*, indicators of anthropogenic forests [11], [19], [29], [39], were found in the same plots as *Astrocaryum murumuru* and *Elaeis oleifera*, both related to ADE [51].

### Understanding the past to conserve and manage for the future

Our study suggests that past human impacts in the forest extend over large areas considered primary forest today, since we found a gradient of human manipulation in the forest from the main rivers into the Purus-Madeira interfluve. Because this interfluve is full of secondary and smaller rivers crossing its interior, the effect of human manipulation should also be directly related to the distance from these rivers. ADE in the vicinity of a secondary river more than 5 km away from the Madeira River contains a unique species composition related to past human activity when compared with non-anthropogenic soils [51]. As most ecological studies focus on the vicinity of the principal navigable rivers [31], these studies need to incorporate the effect of human history to better understand the patterns and mechanisms that explain biodiversity. This issue has been raised previously [52], [53], but is still remarkably ignored by many biologists and ecologists [54]. Future research must associate floristic inventories with paleoecological and archaeological data to build a more complete view of the impact of pre-Colombian populations in Amazonia, especially in other interfluvial areas.

Our results have important implications for the conservation and sustainable use of forest resources today. Although Amazonia is mostly sparsely populated and filled with apparently empty areas today, such as the interfluves, people live in these forests in remote locations. These people, both indigenous and peasant, depend on the forest's resources for their well-being. We argue that the modifications left by ancient Native Amazonians in the landscape and in the useful tree community are extremely important to plan sustainable practices for the use of these forests today [55].

The strategies for Amazonian conservation, suggested by the Brazilian National System of Conservation Units [56], recognize the existence of people living within the forest and extracting non-timber forest products (NTFP). Accordingly, forests that were managed and enriched in the past have an important role for biodiversity conservation, as they concentrate NTFPs that should be sustainably managed by human populations [57]. From this point of view, the role of traditional populations, with their management practices, becomes crucial to ensure the conservation of the forest and the culture of the people who live there.

## Materials and Methods

### Study Area

The study was conducted in the interfluve between the Purus, Madeira and Solimões Rivers, in the state of Amazonas, Brazil (Fig. 1). The study was carried out in mature lowland forests along the BR-319 Highway in six previously installed sites of the Research Program in Biodiversity (PPBio) [58]; for more details about study area and the sites see: Text S1 and http://ppbio.inpa.gov.br/repositorio/dados. The PPBio obtained prior informed consent to install and study all sites. In the PPBio website the identification of each study site from north to south of the interfluve is: M01, M02, M05, M06, M10, M11. We changed the original identification of the sites to produce a more understandable sequence (see Table S2). The sites are located at different distances from the major rivers and in different environments.

### Mapping Archaeological Evidence

Archaeological evidence and anthropogenic forests were identified and mapped around each research site. The main evidence of pre-modern human activities documented were Amazonian Dark Earths (ADE) and two types of anthropogenic forests, the *castanhais* and *caiauezais*. The identification of ADE and anthropogenic forests was obtained by questioning local residents and, when possible, GPS coordinates were recorded on site. After self-presentation and explaining that the project was studying the trees and palms of the local PPBio module, the single question was: where are the ADE, *castanhais* and *caiauezais* near here? Since no information related to use and management of ADE and anthropogenic forest sites was sought from the local residents, the approval of the Ethics Committee for Research with Humans at INPA was considered unnecessary.

### Charcoal Data

We analyzed charcoal in 4–5 plots in each site, for a total of 29 plots surveyed. Two of them are the same plots used for botanical analysis at each site. At the beginning, middle and end of each of the five plots, small pits were excavated to 50 cm in depth. Using a Kopecky cylinder (100 cm^3^), a horizontal collection of undisturbed soil was made at 10 cm intervals. The soil was dried and then visible charcoal was removed for weighing.

### Useful Species

To create the list of useful species considered in this study, we used the most important papers in ethnobotany and previous inventories in anthropogenic forests [4], [11], [39], [41], [46], [51], [59], [60], [61] in Amazonian forests and archeological sites. The useful species mentioned in at least two studies were included in the list. We also considered their degree of domestication [4], their use as food resources in the daily diet of human populations during long periods in the forest for game hunting or other activities, and also their capacity to attract game. Species with commercial value in the post-colonial period, such as *Hevea brasiliensis* and *Carapa guianensis*, were also included in the list.

### Botanical Data

Ten plots were installed in each site by PPBio, one km from each other. In five of them, all trees were marked and measured. After considering the topographic variation between plots, using SRTM (Shuttle Radar Topography Mission) images, we chose two of the five plots to sample tree composition. All trees and palms with diameter at breast height (DBH)≥10 cm were sampled in plots of 0.5 ha (250×20 m). Trees with DBH≥30 cm were sampled in 1 ha plots (250×40 m).

At M4, only two plots had been installed at collection time, and one of them is located less than 500 meters from the highway and on the edge of a shifting cultivation plot. We intended to work only in mature forest, so we excluded this plot from analysis.

We inventoried trees with DBH≥10 cm because they may be descendants of pre-conquest management, since old trees that may have been planted or promoted by ancient people will reproduce and their recruits will persist in old anthropogenic forests. Ross [32] found small individuals of useful tree species (>2.5 cm DBH) in ancient Maya forest gardens after a millennium of abandonment, confirming this expectation.

The botanical material was pre-identified in the field by parataxonomists and also collected for comparison with herbarium collections. After a preliminary identification in the field with the aid of parataxonomists, the botanical identification was confirmed by Priscila Souza, graduate student in Botany at INPA, specialists, identification guides and by comparing the vouchers collected to specimens at the INPA Herbarium (Manaus, Brazil) and virtual herbariums (http://fm1.fieldmuseum.org/vrrc/index.php, http://sciweb.nybg.org/science2/vii2.asp). Fertile specimens were deposited at INPA and sterile material will be deposited at the EAFM Herbarium (Herbarium of the Federal Institution of Amazonas State). Floristic and charcoal data will be available on the PPBio web site and may be requested from the first author.

### Distance Measurements

The distances from botanical plots to rivers were calculated using Landsat Thematic Mapper (TM) images. We considered a straight-line distance from the plot to the closest major river (Solimões, Purus or Madeira). To measure the distance to smaller rivers, a buffer zone with a 25 km radius around each plot was traced. We considered 25 km the maximum distance that could be covered on foot leaving the center of occupation for long hunting activities [47], [48]. Within each buffer, the shortest distance from the plot to perennial rivers (greater than 50 m in width) was calculated. Only 50 m wide rivers were chosen, as this width represents the minimum width of navigable rivers in the region detected using TM images. An index of rivers distances was calculated by the sum of all inverse distance values from each plot to perennial rivers inside the 25 km buffer zone (index of rivers distances = 1−(1/distance river 1+1/distance river 2+1/distance river n)).

### Hydrological Measurements

The gradient of hydrological conditions was measured in two ways. In plots that flooded during the rainy season, we used the height of water marks on tree trunks left by the highest water level in the previous year. In plots that did not flood, we installed a piezometer from the soil surface to seven meters below the ground level. The distance from the soil surface to the highest groundwater level was measured in March 2011, as this is the period with the highest groundwater level during the rainy season.

### Data Analysis

To evaluate the relationship between useful “tree” parameters and the distance to rivers we used simple linear regressions and nonlinear regressions. The useful tree parameters were: 1) the relative abundance of useful species – number of individual useful trees and palms as a percentage of the total number of individuals per plot; 2) the relative richness of useful species – number of useful species as a percentage of the total number of species per plot; and 3) relative basal area occupied by useful species – basal area of useful species as a percentage of the total basal area per plot. We used relative values of abundance, richness and basal area due to the high variation in total numbers from one plot to another (Table 1). The shortest distance from major rivers and the index of rivers distances, which reflect the ability of human movement within the interfluve, were the independent variables used in the regressions. For abundance and basal area parameters, the values of all trees and palms with DBH<30 cm in 0.5 ha were extrapolated to 1 ha.

### Palm Analysis

To determine if the patterns of useful palm abundance observed are associated with environmental parameters, rather than the distance from possible occupation sites, we used multiple regression models. These models included the hydrological gradient as environmental predictor and the distance to rivers as a predictor of human activity, and relative abundance of palms as response variable. The predictor variables had a low Pearson correlation (r = 0.25, p = 0.46).

## Supporting Information

Text S1 Details of the study area.
(DOC)

Text S2 Archaeological evidence and anthropogenic forests.
(DOC)

Table S1 List of useful species found in 11 plots and their respective abundances along the Purus-Madeira interfluve, Amazonas, Brazil.
(DOC)

Table S2 Correspondence between the identification of each study site in the PPBio website and the identification adopted in this study.
(DOC)

## References

[1] Willis KJ, Gillson L, Brncic TM (2004) How “virgin” is virgin rainforest? Science 304: 402–403. 15087539 10.1126/science.1093991

[2] Denevan WM (2011) The “Pristine Myth” revisited. Geogr Rev 101: 576–591.

[3] Piperno DR, Pearsall DM (1998) The Origins of Agriculture in the Lowland Neotropics. San Diego: Academic Press.

[4] Clement CR (1999) 1492 and the loss of Amazonian crop genetic resources. I: The relation between domestication and human population decline. Econ Bot 53: 188–202.

[5] Heckenberger MJ, Kuiruro A, Kuikuro UT, Russel JC, Schmidt M, et al (2003) Amazonia 1492: Pristine forest or cultural parkland? Science 301: 1110–1114. 10.1126/science.1086112 14500979

[6] Erickson CL (2008) Amazonia: The Historical Ecology of a Domesticated Landscape. In: Silverman H, Isbell WH, editors. Handbook of South American Archaeology. Berlin: Springer. pp. 157–183.

[7] Peres CA, Gardner TA, Barlow J, Zuanon J, Michalski F, et al (2010) Biodiversity conservation in human-modified Amazonian forest landscapes. Biol Conserv 143 (10) 2314–2627.

[8] Barlow J, Gardner TA, Lees AC, Parry L, Peres CA (2012) How pristine are tropical forests? An ecological perspective on the pre-Columbian human footprint in Amazonia and implications for contemporary conservation. Biol Conserv 151 (1) 45–49.

[9] Bush MB, Silman MR (2007) Amazonian exploitation revisited: Ecological asymmetry and the policy pendulum. Front Ecol Environ 5: 457–465.

[10] McMichael CH, Bush MB, Piperno DR, Silman MR, Zimmerman AR, et al (2012) Spatial and temporal scales of pre-Columbian disturbance associated with western Amazonian lakes. The Holocene 22 (2) 131–141.

[11] Balée W (1989) The culture of Amazonian forests. In: Posey DA, Balée W, editors. Resource Management in Amazonia: Indigenous and Folk Strategies. New York: The New York Botanical Garden. pp. 1–21.

[12] Denevan WM (1996) A bluff model of riverine settlement in prehistoric Amazonia. Ann Assoc Am Geogr 86 (4) 654–681.

[13] WinklerPrins AMGA, Aldrich SP (2010) Locating Amazon Dark Earths: Creating an interactive GIS of known locations. J Latin Am Geogr 9 (3) 33–50.

[14] Pärssinen M, Schaan D, Ranzi A (2009) Pre-Columbian geometric earthworks in the upper Purús: a complex society in western Amazonia. Antiquity 83: 1084–1095.

[15] Erickson CL, Balee W (2006) The Historical Ecology of a Complex Landscape in Bolivia. In: Balée W, Erickson CL, editors. Time and Complexity in Historical Ecology: Studies in the Neotropical Lowlands. New York: Columbia University Press. pp. 187–233.

[16] Junk WF, Piedade MPF, Schöngart J, Cohn-Haft M, Adeney JM, et al (2011) A classification of major naturally-occurring Amazonian lowland wetlands. Wetlands 31 (4) 623–640.

[17] Clement CR, McCann JM, Smith NJH (2003) Agrobiodiversity in Amazônia and its relationships with Dark Earths. In: Lehmann J, Kern D, Glaser B, Woods W, editors. Amazonian Dark Earths – Origin, Properties, and Management. Dordrecht: Kluwer Academic Publishers. pp. 159–118.

[18] Peters C (2000) Pre-Columbian Silviculture and Indigenous Management of Neotropical Forests. In: Lentz D, editor. Imperfect Balance: Landscape Transformations in the Pre-Columbian Americas. New York: Columbia University Press. pp. 203–223.

[19] Shepard GH, Ramirez H (2011) “Made in Brazil”: human dispersal of the Brazil nut (Bertholletia excelsa, Lecythidaceae) in ancient Amazonia. Econ Bot 65: 44–65.

[20] Chambers JQ, Higuchi N, Schimel JP (1998) Ancient trees in Amazonia. Nature 391: 135–136.

[21] Cunha RNV, Lopes R, Rocha RNC, Lima WAA, Teixeira PC, et al.. (2009) Domesticação e melhoramento de caiaué. In: Borém A, Lopes MTG, Clement CR, editors. Domesticação e Melhoramento: Espécies Amazônicas, Viçosa: Universidade Federal de Viçosa. pp. 275–296.

[22] Posey DA (1985) Indigenous management of tropical forest ecosystems: the case of Kayapó Indians of the Brazilian Amazon. Agrofor Syst 3: 139–158.

[23] Sanford RL Jr, Horn SP (2000) Holocene rain-forest wilderness: A Neotropical perspective on humans as an exotic, invasive species. USDA Forest Serv Proc RMRS 3: 1–15.

[24] Brazil (1978) Projeto RADAMBRASIL Folha SB.20 Purus; geologia, geomorfologia, pedologia, vegetação e uso potencial da terra. Rio de Janeiro: Ministério de Minas e Energia, Departamento Nacional de Produção Mineral.

[25] Moretzsohn MC, Ferreira MA, Amaral ZPS, Coelho PJA, Grattapaglia D, et al (2002) Genetic diversity of Brazilian oil palm (Elaeis oleifera H.B.K.) germplasm collected in the Amazon Forest. Euphytica 124: 35–45.

[26] Piperno DR, Becker P (1996) Vegetation history of a site in the central Amazon Basin derived from phytolith and charcoal records from natural soils. Quat Res 45: 202–209.

[27] Irion G, Mello JASN, Morais J, Piedade MTF, Junk WJ, et al.. (2010) Development of the Amazon Valley During the Middle to Late Quaternary: Sedimentological and Climatological Observations. In: Junk WJ, Piedade MTF, Wittmann F, Schöngart J, Parolin P, editors. Central Amazonian Floodplain Forests: Ecophysiology, Biodiversity and Sustainable Management. Berlin: Springer, Berlin. pp. 27–42.

[28] Guix JC (2005) Evidence of old anthropic effects in forests at the confluence of the Caurés and Negro Rivers – NW Amazonia: The role of Indians and Caboclos. Grupo Estud Ecol Sér Doc 8 (1) 1–27.

[29] Scoles R, Gribel R (2011) Population structure of Brazil nut (Bertholletia excelsa, Lecythidaceae) stands in two areas with different occupation histories in the Brazilian Amazon. Hum Ecol 39: 455–464.

[30] Souza AD, Oliveira RS, Furtado EL, Kageyama PY, Freitas RGS, et al.. (2011) Rubber tree, seringueira (Hevea brasiliensis). In: Fruit Trees and Useful Plants in Amazonian Life. Shanley P, Cymerys M, Serra M, Medina G, editors. Rome: FAO-CIFOR-PPI. pp. 121–128.

[31] Dambrine EJL, Dupouey J-L, Laüt L, Humbert L, Thinon M, et al (2007) Present forest biodiversity patterns in France related to former Roman agriculture. Ecology 88: 1430–1439. 17601136 10.1890/05-1314

[32] Ross NJ (2011) Modern tree species composition reflects Ancient Maya ‘forest gardens’ in NW Belize. Ecol Appl 21 (1) 75–84. 21516889 10.1890/09-0662.1

[33] van Gemerden BS, Olff H, Parren MPE, Bongers F (2003) The pristine rain forest? Remnants of historical human impacts on current tree species composition and diversity. J Biogeogr 30: 1381–90.

[34] Pitman NCA, Widmer J, Jenkins CN, Stocks G, Seales L, et al (2011) Volume and geographical distribution of ecological research in the Andes and the Amazon, 1995–2008. Trop Conserv Sci 4: 64–81.

[35] Smith NJH (1980) Anthrosols and human carrying capacity in Amazonia. Ann Assoc Am Geogr 70 (4) 553–566.

[36] Kahn F, Mejia K (1990) Palm communities in wetland forest ecosystems of Peruvian Amazonia. For Ecol Manage 33/34: 169–119.

[37] Wittman F, Junk WJ, Piedade MTF (2004) The varzea forests in Amazônia: flooding and the highly dynamic geomorphology interact with natural forest succession. Forest Ecol Manag 196 (2–3) 199–212.

[38] Kahn F, Castro A (1985) The palm community in a forest of central Amazonia, Brazil. Biotropica 11: 210–216.

[39] Balée W, Campbell DG (1990) Evidence for the successional status of liana forest (Xingu River basin, Amazonian Brazil). Biotropica 22: 36–47.

[40] Morcote-Rios G, Bernal R (2001) Remains of palms (Palmae) at archaeological sites in the new world: a review. Bot Rev 67: 309–350.

[41] Cavalcante PB (2010) Frutas Comestíveis na Amazônia. Belém: Museu Paraense Emílio Goeldi.

[42] Salm R (2005) The importance of forest disturbance for the recruitment of the large arborescent palm Attalea maripa in a seasonally-dry Amazonian forest. Biota Neotrop 5 (1) 1–7.

[43] Svenning JC (1999) Recruitment of tall arborescent palms in the Yasuni National Park, Amazonian Ecuador: are large treefall gaps important? J Trop Ecol 15: 355–366.

[44] Bush MB, Silman MR, Toledo MB, Listopad C, Gosling WD, et al (2007) Holocene fire and occupation in Amazonia: records from two lake districts. Philos Trans R Soc Biol Sci 362: 209–218. 10.1098/rstb.2006.1980 PMC2311425 17255030

[45] McMichael CH, Piperno DR, Bush MB, Silman MR, Zimmerman AR, et al (2012) Sparse pre-Columbian human habitation in Western Amazonia. Science 336: 1 429–1431. 10.1126/science.1219982 22700926

[46] López-Zent E, Zent S (2004) Amazonian Indians as Ecological Disturbances Agents: The Hotï of the Sierra Maigualida, Venezuelan Guayana. In: Carlson TS, Maffi L, editors. Ethnobotany and Conservation of Biocultural Diversity. New York: The New York Botanical Garden. pp. 79–112.

[47] Shepard G (2002) Primates in Matsigenka subsistence and world view. In: Fuentes A, Wolfe LD, editors. Primates face to face: conservation implications of human and nonhuman primates interconnections. Cambridge: Cambridge University Press. pp. 101–136

[48] Peres CA, Nascimento HS (2006) Impact of game hunting by the Kayapo of south-eastern Amazonia: implications for wildlife conservation in tropical forest indigenous reserves. Biodiv Conserv 15: 2627–2653.

[49] Politis GG (1996) Moving to produce: Nukak mobility and settlement patterns in Amazonia. World Archaeology 27: 492–511.

[50] Stahl PW (2008) The contributions of zooarchaeology to historical ecology in the Neotropics. Quaternary International 180: 5–16.

[51] Junqueira AB, Shepard GH Jr, Clement CR (2010) Secondary forests on anthropogenic soils in Brazilian Amazonia conserve agrobiodiversity. Biodiv Conserv 19: 1933–1961.

[52] Clark DB (1996) Abolishing virginity. J Trop Ecol 12 (5) 735–739.

[53] Wills KJ, Araújo MB, Bennett KD, Figueroa-Rangel B, Froyd CA, et al (2007) How can a knowledge of the past help to conserve the future? Biodiversity conservation and the relevance of long-term ecological studies. Phil Trans R Soc B 362: 115–186. 10.1098/rstb.2006.1977 PMC2311423 17255027

[54] Clement CR, Junqueira AB (2010) Between a pristine myth and an impoverished future. Biotropica 42: 534–536.

[55] Heckenberger MJ, Russell JC, Toney JR, Schmidt MJ (2007) The legacy of cultural landscapes in the Brazilian Amazon: Implications for biodiversity. Phil Trans R Soc B 362: 197–208. 17255029 10.1098/rstb.2006.1979 PMC2311456

[56] Brasil (2000) Sistema Nacional de Unidades de Conservação da Natureza – SNUC. Brasília: Diario Oficial da União.

[57] Scoles R, Gribel R (2012) The regeneration of Brazil nut tree in relation to nut harvest intensity in the Trombetas River valley of Northern Amazonia, Brazil. For Ecol Manage 265: 71–81.

[58] Magnusson B, Costa F, Lima A, Baccaro F, Braga-Neto R, et al (2008) A program for monitoring biological diversity in the Amazon: an alternative perspective to threat-based monitoring. Biotropica 40: 409–411.

[59] Balée W (2010) Contingent diversity in anthropic landscapes. Diversity 2: 163–181.

[60] Junqueira AB, Shepard GH, Clement CR (2011) Secondary forests on anthropogenic soils of the Middle Madeira River: Valuation, local knowledge, and landscape domestication in Brazilian Amazonia. Econ Bot 65: 85–99.

[61] Prance GT, Balée W, Boom BM, Carneiro RL (1987) Quantitative ethnobotany and the case for conservation in Amazonia. Conserv Biol 1 (2) 296–310.

