# Supplementary material for: Historical Human Footprint on Modern Tree Species Composition in the Purus-Madeira Interfluve, Central Amazonia
Source: PLoS One. 2012 Nov 20;7(11):e48559. doi: 10.1371/journal.pone.0048559 (PMC3502455; doi:10.1371/journal.pone.0048559)
Supplement: Text S1 — Details of the study area. (DOC) [file pone.0048559.s001.doc]

**Supplementary Text S1: Detail of the study area**

**Study Area**. The region is characterized by very flat topography [1]. M1, M2 and M6 are on *paleo-várzeas,* old floodplains formed during previous glacial periods [1,2]. The other sites are on low plateaus [1]. The soils are mostly Plinthosols (FAO soil taxonomy), with hydromorphic soils on the terraces and alluvial plains [3]. Most soils are poorly drained during the rainy season, causing flooding. Rainfall is high, ranging from 2300 mm in the southern portion of the interfluve to 2750 mm in the north [1]. There is an increase in seasonality from one to three months with less than 100 mm of rain towards the south [4]. The vegetation is defined as Dense Lowland Rainforest in the north and Open Lowland Rainforest in the south [5]. The BR-319 Highway, which connects the cities of Manaus and Porto Velho, is the only road access to the interfluve, allowing the placement of sites in areas far from the major rivers.

**History of human presence in the region**. During the pre-colonial period, the main indigenous ethnic group mentioned as inhabitants of the northern part of the region was the Mura [6]. With the arrival of Europeans, disputes over the territory and diseases resulted in the deaths of most indigenous people [7]. In the late nineteenth century, the Madeira River was reoccupied for the production of latex extracted from the rubber tree (*Hevea brasiliensis*); the rubber boom collapsed in 1920. Fifty years later, the region experienced a new process of population expansion with the construction of the BR-319 Highway, allowing a movement of migrants into the interfluve. Currently, the road is almost impassable in most parts (only the northern and southern sections are used regularly), and most people live in cities and small towns near rivers. However, the BR-319 road is being repaved. Consequently, the region will experience another expansion process.

Kern *et al*. [8] report some patches of Amazonian Dark Earth (ADE) along the Purus River. Most of them are present in the southern portion of this river’s basin. However, the lower Purus, where it empties into the Solimões River, is part of the largest known center of past human occupation in Central Amazonia [9]. On the banks of the Madeira River and its tributaries, a large number of archaeological sites with ADE were also found [10]. Furthermore, the lower Madeira River also has high crop genetic diversity [11].

**References**

1. Brazil (1978) Projeto RADAMBRASIL Folha SB.20 Purus; geologia, geomorfologia, pedologia, vegetação e uso potencial da terra. Rio de Janeiro: Ministério de Minas e Energia, Departamento Nacional de Produção Mineral.

2. Irion G, Mello JASN, Morais J, Piedade MTF, Junk WJ, et al. (2010) Development of the Amazon Valley During the Middle to Late Quaternary: Sedimentological and Climatological Observations. In: Junk WJ, Piedade MTF, Wittmann F, Schöngart J, Parolin P, editors. Central Amazonian Floodplain Forests: Ecophysiology, Biodiversity and Sustainable Management. Berlin: Springer, Berlin. pp. 27-42.

3. Quesada CA, Lloyd J, Anderson LO, Fyllas NM, Schwarz M, et al. (2011) Soils of Amazonia with particular reference to the RAINFOR sites. Biogeosciences 8: 1415–1440.

4. Sombroek W (2001) Spatial and temporal patterns of Amazon rainfall. Ambio 30: 388-

396.

5. IBGE (1997) Recursos Naturais e Meio Ambiente: uma visão do Brasil. Brasília: Instituto Brasileiro de Geografia e Estatística.

6. Cunha MMLC (1992) História dos Índios no Brasil. São Paulo: Companhia das Letras.

7. O'Fallon BD, Fehren-Schmitz L (2011) Native Americans experienced a strong population bottleneck coincident with European contact. Proc Natl Acad Sci USA 108(51): 20444-20448.

8. Kern D, D’Aquino G, Rodrigues TE, Frazão FJL, Sombroek W, et al. (2003) Distributions of Amazonian Dark Earths in the Brazilian Amazon. In: Lehmann J, Kern D, Glaser B, Woods W, editors. Amazonian Dark Earths ‐ Origin, Properties and Management. Dordrecht: Kluwer Academics Publisher. pp. 51‐75.

9. Neves EG, Petersen JB (2006) Political Economy and Pre-Columbian Landscape Transformations in Central Amazonia. In: Balée W, Erickson CL, editors. Time and Complexity in Historical Ecology: Studies in the Neotropical Lowlands. New York: Columbia University Press. pp. 279–309.

10. WinklerPrins AMGA**,** Aldrich SP (2010) Locating Amazon Dark Earths: Creating an interactive GIS of known locations. J Latin Am Geogr 9(3): 33-50.

11. Clement CR (1999) 1492 and the loss of Amazonian crop genetic resources. I: The relation between domestication and human population decline. Econ Bot 53: 188-202.
