# Supplementary material for: Historical Human Footprint on Modern Tree Species Composition in the Purus-Madeira Interfluve, Central Amazonia
Source: PLoS One. 2012 Nov 20;7(11):e48559. doi: 10.1371/journal.pone.0048559 (PMC3502455; doi:10.1371/journal.pone.0048559)
Supplement: Text S2 — Archaeological evidence and anthropogenic forests. (DOC) [file pone.0048559.s002.doc]

**Supplementary Text S2: Archaeological evidence and anthropogenic forests**

The M1 site had the highest number and diversity of evidence (Fig. 1). *Castanhais* and *caiauézais* were close to the plots (Table 1). ADE was only 4 km from the plots and was located in a soil susceptible to flooding in years with very intense floods. M2 was close to one *castanhal* and the Janauacá River. Local residents affirm that there are many ADE sites around Janauacá Lake, but they have not yet been mapped. At M3 and M4, ADE was mapped more than 10 km from the sites at the Igapó Açu River, which is the longest river in the interfluve. At M5, *castanhais* were mapped close to the plot at the edge of a watercourse connected with the Acará River. According to local residents, this river was occupied by Mura Indians in the past and numerous Brazil nut stands occur on its banks. No ADE was mapped near M6, but this was the only site with Brazil nut trees, *caiaué* and other domesticated plants within the sampled plots (Supplementary Table S1).

**Useful species.** We found 34 useful species from 14 different botanical families in 11 plots. The highest abundance of useful species was found in one plot of M1, the closest to a major river, the Solimões, and the lowest abundance was found at M4, located in an upland forest, 80 km from the Madeira River (Table 1). The five useful species with the largest number of individuals in M1 were *Euterpe precatoria, Hevea brasiliensis, Carapa guianensis, Anacardium parvifolium* and *Symphonia globulifera* ; in M6, *Attalea speciosa, Euterpe precatoria, Astrocaryum murumuru, Oenocarpus bataua* and *Pseudolmedia laevis*; and in M2, *Oenocarpus bataua, Tapirira guianensis Micropholis guyanensis, Helicostylis tomentosa* and *Theobroma* spp. (Supplementary Table S1). These three sites have different useful dominant species.
