# Supplementary material for: Historical Human Footprint on Modern Tree Species Composition in the Purus-Madeira Interfluve, Central Amazonia
Source: PLoS One. 2012 Nov 20;7(11):e48559. doi: 10.1371/journal.pone.0048559 (PMC3502455; doi:10.1371/journal.pone.0048559)
Supplement: Table S1 — List of useful species found in 11 plots and their respective abundances along the Purus-Madeira interfluve, Amazonas, Brazil. (DOC) [file pone.0048559.s003.doc]

Table S1. List of useful species found in 11 plots and their respective abundances along the Purus-Madeira interfluve, Amazonas, Brazil

| **Species** | **Family** | **Common name** | **Degree of domestication*** | **M1.1** | **M1.1** | **M2.1** | **M2.2** | **M3.1** | **M3.2** | **M4** | **M5.1** | **M5.2** | **M6.1** | **M6.2** |
| --- | --- | --- | --- | --- | --- | --- | --- | --- | --- | --- | --- | --- | --- | --- |
| *Anacardium parvifolium* Ducke | Anacardiaceae | cajuí | - | 8 | 4 | 0 | 0 | 0 | 0 | 0 | 0 | 0 | 0 | 0 |
| *Astrocaryum aculeatum* G.Mey. | Arecaceae | tucumã | semi-domesticated | 0 | 0 | 0 | 0 | 0 | 0 | 0 | 0 | 0 | 8 | 0 |
| *Astrocaryum murumuru* Mart. | Arecaceae | muru-muru | incipiently | 2 | 4 | 4 | 0 | 0 | 0 | 0 | 0 | 0 | 0 | 18 |
| *Attalea speciosa* Mart. ex Spreng. | Arecaceae | babaçú | - | 2 | 0 | 0 | 0 | 0 | 0 | 0 | 8 | 12 | 70 | 35 |
| *Attalea maripa* (Aubl.) Mart. | Arecaceae | inajá | incipiently | 0 | 0 | 6 | 6 | 2 | 2 | 0 | 0 | 0 | 0 | 0 |
| *Bertholletia excelsa* Bonpl. | Lecythidaceae | castanheira | incipiently | 0 | 0 | 0 | 0 | 0 | 0 | 0 | 0 | 0 | 10 | 2 |
| *Carapa guianensis* Aubl. | Meliaceae | andiroba | - | 9 | 23 | 0 | 0 | 1 | 2 | 0 | 4 | 0 | 0 | 0 |
| *Caryocar glabrum* (Aubl.) Pers. | Caryocaraceae | pequiarana | incipiently | 0 | 0 | 0 | 0 | 0 | 0 | 2 | 1 | 4 | 4 | 4 |
| *Copaifera multijuga* Hayne | Fabaceae | copaiba | - | 0 | 0 | 0 | 0 | 0 | 2 | 0 | 2 | 6 | 0 | 2 |
| *Couepia guianensis* Aubl. | Chrysobalanaceae | pajurá | - | 0 | 0 | 0 | 0 | 0 | 0 | 0 | 0 | 0 | 10 | 0 |
| *Couma macrocarpa*Barb.Rod. | Apocynaceae | sorva | - | 0 | 0 | 0 | 3 | 2 | 0 | 4 | 5 | 0 | 0 | 0 |
| *Dipteryx odorata* (Aubl.) Willd. | Fabaceae | cumarú | - | 0 | 0 | 0 | 0 | 2 | 3 | 0 | 0 | 0 | 0 | 3 |
| *Ecclinusa guianensis* Eyma | Sapotaceae | guajaraí | - | 0 | 0 | 0 | 2 | 0 | 13 | 2 | 2 | 7 | 2 | 7 |
| *Elaeis oleifera* (Kunth) Cortés | Arecaceae | caiaué | incipiently | 0 | 0 | 0 | 0 | 0 | 0 | 0 | 0 | 0 | 0 | 2 |
| *Endopleura uchi*(Huber) Cuatrec. | Humiriaceae | uxí | - | 0 | 0 | 0 | 0 | 4 | 0 | 2 | 0 | 1 | 1 | 0 |
| *Euterpe precatoria* Mart. | Arecaceae | açaí | - | 102 | 157 | 6 | 0 | 0 | 0 | 0 | 20 | 12 | 8 | 12 |
| *Garcinia sp.1* L. | Clusiaceae | bacuri | - | 0 | 0 | 0 | 0 | 0 | 4 | 0 | 0 | 0 | 0 | 0 |
| *Helicostylis tomentosa* (Poepp. & Endl.) Rusby | Moraceae | inharé | - | 0 | 0 | 4 | 14 | 0 | 7 | 10 | 12 | 5 | 0 | 0 |
| *Hevea brasiliensis* (Willd. ex A.Juss.) Müll.Arg. | Euphorbiaceae | seringueira | incipiently | 7 | 30 | 0 | 0 | 6 | 13 | 0 | 2 | 3 | 0 | 0 |
| *Hymenaea parvifolia* Huber | Fabaceae | jutaí | - | 10 | 0 | 0 | 0 | 0 | 0 | 0 | 0 | 0 | 0 | 0 |
| *Inga gracilifolia* Ducke | Fabaceae | inga | - | 7 | 0 | 0 | 2 | 2 | 0 | 0 | 0 | 0 | 0 | 0 |
| *Inga alba* (Sw.) Willd. | Fabaceae | inga | - | 0 | 4 | 0 | 0 | 1 | 0 | 0 | 0 | 0 | 0 | 0 |
| *Manilkara bidentata* (A.DC.) A.Chev. | Sapotaceae | massaranduba | - | 0 | 0 | 0 | 0 | 0 | 3 | 0 | 6 | 2 | 0 | 0 |
| *Micropholis guyanensis* (A.DC.) Pierre | Sapotaceae | balata | - | 0 | 0 | 2 | 19 | 0 | 10 | 9 | 7 | 7 | 0 | 0 |
| *Oenocarpus bacaba* Mart. | Arecaceae | bacaba | incipiently | 0 | 0 | 0 | 6 | 20 | 4 | 8 | 4 | 0 | 0 | 0 |
| *Oenocarpus bataua* Mart. | Arecaceae | patauá | incipiently | 0 | 0 | 94 | 46 | 0 | 10 | 0 | 0 | 0 | 2 | 16 |
| *Parinari excelsa* Sabine | Chrysobalanaceae | pajurá | - | 0 | 0 | 3 | 0 | 0 | 0 | 1 | 0 | 0 | 0 | 10 |
| *Pseudolmedia laevis* (Ruiz & Pav.) J.F.Macbr. | Moraceae | inharé | - | 0 | 0 | 6 | 6 | 4 | 16 | 2 | 0 | 0 | 17 | 0 |
| *Symphonia globulifera* L.f. | Clusiaceae | bacuripari | - | 8 | 4 | 2 | 0 | 2 | 5 | 0 | 7 | 0 | 0 | 6 |
| *Tapirira guianensis* Aubl. | Anacardiaceae | tapiriri | - | 0 | 0 | 15 | 5 | 6 | 0 | 2 | 0 | 0 | 0 | 10 |
| *Theobroma obovatum* Klotzsch ex Bernoulli | Malvaceae | cacaurana | - | 4 | 6 | 0 | 0 | 0 | 0 | 0 | 0 | 0 | 6 | 0 |
| *Theobroma speciosum* Willd. ex Spreng. | Malvaceae | cacaurana | incipiently | 0 | 0 | 0 | 3 | 2 | 0 | 0 | 0 | 0 | 0 | 0 |
| *Theobroma subincanum* Mart. | Malvaceae | cupuí | incipiently | 0 | 0 | 0 | 4 | 2 | 0 | 0 | 0 | 0 | 0 | 0 |
| *Theobroma sylvestre* Mart. | Malvaceae | cacau-do-mato | - | 0 | 0 | 2 | 2 | 0 | 0 | 0 | 0 | 0 | 0 | 0 |

*The degree of domestication according to the classification of Clement [1].

**Reference:**

1. Clement CR (1999) 1492 and the loss of Amazonian crop genetic resources. I: The relation between domestication and human population decline. Econ Bot 53: 188-202.
