# Supplementary material for: Historical Human Footprint on Modern Tree Species Composition in the Purus-Madeira Interfluve, Central Amazonia
Source: PLoS One. 2012 Nov 20;7(11):e48559. doi: 10.1371/journal.pone.0048559 (PMC3502455; doi:10.1371/journal.pone.0048559)
Supplement: Table S2 — Correspondence between the identification of each study site in the PPBio website and the identification adopted in this study. (DOC) [file pone.0048559.s004.doc]

**Table S2. Correspondence between the identification of each study site in the PPBio website and the identification adopted in this study**

PPBio website M01 M02 M05 M06 M10 M11

Our study M1 M2 M3 M4 M5 M6
